# Bioinspired, roughness-induced, water and oil super-philic and super-phobic coatings prepared by adaptable layer-by-layer technique

**DOI:** 10.1038/srep14030

**Published:** 2015-09-10

**Authors:** Philip S. Brown, Bharat Bhushan

**Affiliations:** 1 Nanoprobe Laboratory for Bio- & Nanotechnology and Biomimetics (NLBB), The Ohio State University, 201 W. 19th Avenue, Columbus, OH 43210-1142, USA

## Abstract

Coatings with specific surface wetting properties are of interest for anti-fouling, anti-fogging, anti-icing, self-cleaning, anti-smudge, and oil-water separation applications. Many previous bioinspired surfaces are of limited use due to a lack of mechanical durability. Here, a layer-by-layer technique is utilized to create coatings with four combinations of water and oil repellency and affinity. An adapted layer-by-layer approach is tailored to yield specific surface properties, resulting in a durable, functional coating. This technique provides necessary flexibility to improve substrate adhesion combined with desirable surface chemistry. Polyelectrolyte binder, SiO_2_ nanoparticles, and silane or fluorosurfactant layers are deposited, combining surface roughness and necessary chemistry to result in four different coatings: superhydrophilic/superoleophilic, superhydrophobic/superoleophilic, superhydrophobic/superoleophobic, and superhydrophilic/superoleophobic. The superoleophobic coatings display hexadecane contact angles >150° with tilt angles <5°, whilst the superhydrophobic coatings display water contact angles >160° with tilt angles <2°. One coating combines both oleophobic and hydrophobic properties, whilst others mix and match oil and water repellency and affinity. Coating durability was examined through the use of micro/macrowear experiments. These coatings display transparency acceptable for some applications. Fabrication via this novel combination of techniques results in durable, functional coatings displaying improved performance compared to existing work where either durability or functionality is compromised.

The surface properties of a coating, with regards to wetting by liquids, are determined by the chemistry and topography of the interface. For a flat surface, a liquid droplet will rest on the surface with a contact angle determined by a combination of interfacial tensions^1^


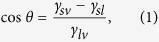


where γ_*sv*_, γ_*sl*_, and γ_lv_** are the solid–vapor, solid–liquid, and liquid–vapor surface tensions respectively, and *θ* is the contact angle of the droplet. When a surface is roughened, the surface properties can be changed due to an amplification of the solid–liquid interactions, assuming the liquid fully wets the surface^2^. It is also possible for air pockets to become trapped between the solid and the liquid resulting in a composite interface^3^ and, since the liquid is resting partially on air, a more repellent surface.

By selecting the correct chemistry and topography, a coating can display a variety of liquid wetting properties. For a review of the literature, see Table 1. These properties can be exploited for a variety of applications. For instance, coatings that repel water (hydrophobic) are useful for self-cleaning applications^4^. In nature, this is most evident in the lotus leaf 5; the superhydrophobic properties of the leaf surface, achieved through the presence of hierarchical structure created by rough papillae and superimposed with hydrophobic wax nanotubules, cause water droplets to roll around the surface of the leaf, collecting contaminants as they go thus keeping the leaf clean^5^.

Coatings that attract water (hydrophilic) are useful for anti-fogging applications^6^; any liquid water spreads out into a thin film thereby maintaining transparency. This is more favorable than using hydrophobic surfaces for anti-fogging as this requires a surface to be tilted for the droplets to roll off and transparency be maintained. Hydrophilic surfaces can also be used for self-cleaning^7^. Previous examples of superhydrophilic surfaces include the use of polymer–nanoparticle coatings^8^,^9^,^10^,^11^ however mechanical durability was not investigated.

Coatings with surface tensions lower than that of water (72 mN m^–1^) but higher than that of oils^12^ (20–30 mN m^–1^) will attract oils (oleophilic) but repel water and can be used to create oil–water separators^13^,^14^,^15^. When applied to a porous substrate, the coating will allow the passage of oil but block the passage of water, resulting in their separation. In addition, their water repellency also makes them ideal for self-cleaning^4^,^16^ and anti-icing^17^,^18^,^19^ applications. Anti-icing surfaces are typically superhydrophobic as supercooled droplets of water are able to roll off the cold surface before freezing and any ice formed is weakly adhered compared to hydrophilic surfaces due to an air cushion^18^,^20^.

Coatings with lower surface tensions (∼20 mN m^–1^ or less) will repel both oil (oleophobic) and water and are useful for anti-fouling such as in medical and transport applications, where both the oil-repellency and nanostructuring are of importance^21^,^22^,^23^,^24^,^25^,^26^,^27^. Previous work was not suitable for such applications as either the durability^28^ or oil-repellency^29^ was not optimal. The oil repellency also makes these surfaces ideal for anti-smudge applications^30^,^31^ where the oils from fingers are not deposited onto the surface and the surface remains clear. The water repellency means these coatings can also be used in self-cleaning and anti-icing applications.

Surfaces that repel oils typically also repel water. This is due to the fact that water has a higher surface tension than oils (Eq. 1). However, it is possible to create a coating that repels oils but attracts water^32^,^33^,^34^,^35^,^36^,^37^,^38^,^39^,^40^,^41^. This is usually achieved through the use of a fluorosurfactant^32^,^33^,^35^,^36^,^37^,^38^,^39^,^40^. A fluorosurfactant contains a high surface tension head group and a low surface tension tail group. When deposited onto a surface, the fluorinated tails segregate at the air interface resulting in a low surface tension barrier that repels oils. However, when droplets of water are placed on such a surface, they are able to penetrate down through the tail groups to reach the high surface tension polar head groups below^42^, and thus the coating appears hydrophilic. Figure 1 schematically compares this so called “flip-flop” of surface properties to that of a typical, “non–flip-flop”, case where penetration does not occur. However, many current examples of “flip-flop” superhydrophilic/superoleophobic surfaces have several drawbacks including poor oleophobicity^36^,^37^,^38^ or poor penetration by water resulting in a coating that is initially hydrophobic^34^,^38^,^39^.

Such a “flip-flop” of surface properties can be exploited for a variety of applications, such as oil–water separation and anti-fouling. In fact, superhydrophilic/superoleophobic oil–water separators are favorable compared to more traditional superhydrophobic/superoleophilic separators as water is denser than oil and tends to sink to the bottom of a mixture. Additionally, oleophilic separators or absorbent materials can quickly become fouled by oil and oil based contaminants requiring cleaning or replacement^43^. Superhydrophilic/superoleophobic separators have no such issue since the oil phase is the one being repelled.

There are various existing methods for fabrication of coatings with different surface properties, Table 1. Most typically use a “one-pot” technique where all the materials are mixed and deposited together. Such a technique can lead to a coating with poor durability as the (typically low surface tension) material used to achieve the desired surface properties is distributed throughout the coating. In fact, many of the previous studies do not report any durability data. In addition, each surface property requires different materials and methods and there is not yet a single method that can achieve all four combinations of water and oil repellency and affinity.

One deposition technique that is not “one-pot” instead utilizes interactions between charged components and is known as the “layer-by-layer” technique, where components are kept separate and deposited individually. Layers of oppositely charged species are deposited one after another to create a multi-layer coating bound together through electrostatic interactions. Many different charged species can be utilized when creating the layered coating and therefore the technique is highly flexible and has been used in a variety of applications. For example, the technique has been used for the creation of a layered microreactor^44^, ultrathin films of conducting polymers^45^, and superhydrophilic surfaces^9^,^11^. Due to the use of water-soluble polymers, layer-by-layer coatings are typically hydrophilic and oleophilic. However, via deposition of a final layer, the functionality of the coating can be altered without compromising the adhesion of the coating.

In this paper, we investigate a fabrication method that can easily be adapted to produce a coating that displays the four possible combination of water and oil repellence or affinity. This is achieved through the use of an adapted layer-by-layer technique. The coating is comprised of several discrete layers, which are deposited individually. A nanoparticle layer introduces roughness to enhance the surface properties of the functional layer and increase the hardness of the coating to improve durability. Intermediate layers are used to help bind the particles to the surface. The final (top) layer in the coating contains the desired surface chemistry and can be easily swapped to produce a different functionality as shown in Table 2. This ensures that the desired functionality is only present at the solid–air interface and not distributed throughout the coating (as in “one-pot” techniques) where it may compromise adhesion and durability. Durability is important if these coatings are to be feasible for application in various industries including medical, transportation, aerospace, energy, and construction.

By using this adapted layer-by-layer technique, it is possible to fabricate coatings with the four possible combinations of water and oil repellency and affinity. In the case of the superhydrophilic/superoleophilic coating, no additional functional layer is added to leave the high surface tension polymer layer exposed. For superhydrophobic/superoleophilic coatings, a non-fluorinated silane is used to repel water but not oils, which have lower surface tensions (surface tension 20–30 mN m^–1^). For superhydrophobic/superoleophobic coatings, a fluorinated silane is used to repel both water and oils. Finally, for superhydrophilic/superoleophobic coatings, a fluorosurfactant is used to yield the desired “flip-flop” of surface properties required. In all cases, the inclusion of a nanoparticle layer enhances the surface properties of the functional layer to result in super-philic/super-phobic surfaces.

We believe this is the first time a single, facile fabrication method has been shown to result in all four possibilities of water and oil repellency and affinity. The durability and functionality of all the coatings have been tested for a variety of applications including anti-fouling, anti-fogging, anti-icing, self-cleaning, anti-smudge, and oil–water separation. We have previously reported the superhydrophilic/superoleophobic^46^ and superhydrophobic/superoleophobic^47^ coatings produced via this technique. The results are included here for completeness. In addition, we have performed additional experimentation, including anti-fogging and anti-icing, on these coatings to further assess their versatility.

## Experimental details

**Figure 1:**
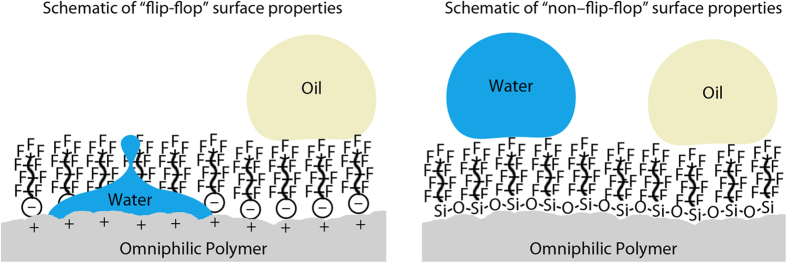
Schematic of “flip-flop” vs “non–flip-flop” surface properties. For the “flip-flop” coating, water is able to penetrate down through the repellent surfactant tails of the functional layer (fluorosurfactant) to the high surface tension portion of the coating while the bulky oil molecules are repelled. For non–flip-flop coatings, water is unable to penetrate the functional layer (fluorosilane).

**Figure 2:**
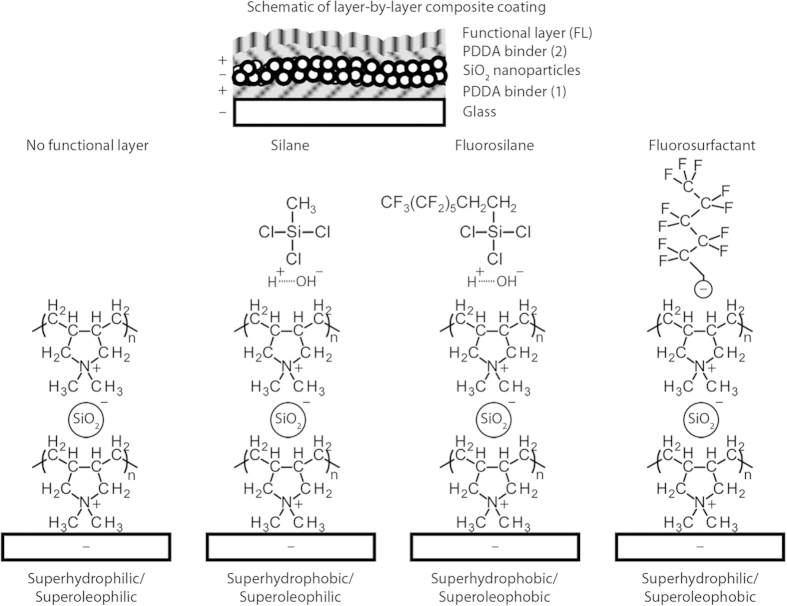
Schematic of the four layer-by-layer composite coatings. Each layer is deposited separately. Also shown are the chemical composition and charge of each layer. The functional layer (FL) is deposited last and provides the desired surface chemistry.

Each coating described in this paper comprises various layers as shown in Fig. 2, deposited separately, each of which aids the creation of a mechanically durable, functional coating. As previously noted^46^,^47^, PDDA was chosen as the polymer base layer as it has a high cationic charge density and has been shown to bind strongly to glass substrates^11^,^48^ and SiO_2_ nanoparticles. The specific molecular weight range (100,000–200,000) was chosen to balance mechanical properties and ease of deposition (viscosity). Untreated, hydrophilic SiO_2_ nanoparticles were used to enhance the roughness of the coating. The negatively charged surface silanol groups ensure good adhesion to the positively charged polymer layers. Additionally, SiO_2_ nanoparticles are known to have high hardness^49^ and wear resistance^50^, which will aid in the creation of a mechanically durable coating^51^. Particles of 7 nm in diameter were selected with the goal to create a transparent coating. The material selected for the final, functional layer varied depending upon the desired surface properties. For the superhydrophilic/superoleophilic coating, no additional layer was deposited. For the superhydrophobic/superoleophilic and superhydrophobic/superoleophobic coatings, two different silanes (non-fluorinated and fluorinated silanes respectively) were selected to provide the desired repellency and because of their ability to form self-assembled layers via vapor phase deposition. Silanes have been shown to condense on hydrophilic polymer layers in the past due to the presence of absorbed water^52^. Finally, for the superhydrophilic/superoleophobic coating (“flip-flop” coating, Fig. 1), a fluorosurfactant was selected for its oil repellency (low surface tension tail) and its ability to complex to a positively charged polyelectrolyte (high surface tension head group).

### Samples

Glass slides (Fisher Scientific) cut to dimensions of 25 by 10 mm were used as substrates. Polydiallyldimethylammonium chloride (PDDA, MW 100,000–200,000, Sigma Aldrich) was dissolved in distilled water (DS Waters of America Inc.) at a concentration of 15 mg mL-1. Silica nanoparticles (NP, 7 nm diameter, Aerosil 380, Evonik Industries) were dispersed in acetone (Fisher Scientific Inc.) using an ultrasonic homogenizer (Branson Sonifier 450A, 20 kHz frequency at 35% amplitude) at various concentrations. The fluorosurfactant solution (FL, Capstone FS-50, DuPont) was diluted with ethanol (Decon Labs Inc) so that the overall fluorosurfactant concentration was 45 mg mL^–1^. Coatings were deposited via spray gun (Paasche) operated with compressed air at 210 kPa. The gun was held 10 cm from the glass slide at all times. First, PDDA solution (52 mg mL^–1^, 2 mL) was spray coated and any excess was removed from the surface via bursts of compressed air from the spray gun. Second, the SiO_2_ NP solution (15 mg mL-1, 3 mL) was spray coated. Third, a second PDDA layer was deposited (8 mg mL^–1^, 1 mL). After this, the samples were transferred to an oven operating at 140 °C for 1 h. Finally, the functional layer (FL) was deposited either via spray coating or chemical vapor deposition under atmospheric conditions. For spray coating, the fluorosurfactant solution (1 mL) was spray coated and the samples were allowed to dry in air. For chemical vapor deposition, one drop of either methyltrichlorosilane (methylsilane, Sigma Aldrich) for superhydrophobic/superoleophilic coatings or trichloro(1H,1H,2H,2H-perfluorooctyl) silane (fluorosilane, Sigma Aldrich) for superhydrophobic/superoleophobic coatings was deposited next to the samples which were covered and left for 6 h.

### Contact angle and tilt angle

For contact angle data, 5-μL droplets of water and n-hexadecane (99%, Alfa Aesar) were deposited onto samples using a standard automated goniometer (Model 290, Ramé-Hart Inc.) and the resulting image of the liquid–air interface analyzed with DROPimage software. Tilt angles were measured by inclining the surface until the 5 μL droplet rolled off. Contact angle hysteresis was measured by tilting the substrate until the droplet was observed to move and the advancing and receding angles were recorded. These numbers were found to be comparable to the tilt angles and are not reported. All angles were averaged over at least five measurements on different areas of a sample.

### Coating thickness

The coating thickness of each individual layer and the composite coating was measured with a step technique. One half of the substrate was covered with a glass slide using double-sided sticky tape before coating and then removed after the coating procedure resulting in a step. An area including the step was imaged using a D3000 Atomic Force Microscopy (AFM) with a Nanoscope IV controller (Bruker Instruments) to obtain the coating thickness. A Si, n-type (Si_3_N_4_) tip with an Al coating (resonant frequency f = 66 kHz, spring constant k = 3 N m^–1^, AppNano) operating in tapping mode was used.

### Wear experiments

The mechanical durability of the surfaces was examined through wear experiments using an AFM and a ball-on-flat tribometer^53^. An established AFM micro-wear procedure was performed with a commercial AFM (D3000, Nanoscope IV controller, Bruker Instruments). Surfaces were worn using a borosilicate ball with radius 15 μm mounted on a rectangular cantilever with nominal spring constant of 7.4 N m^–1^ (resonant frequency f = 150 kHz, All-In-One). Areas of 50 × 50 μm^2^ were worn for 1 cycle at a load of 10 μN so as to be later imaged within the scanning limits of the AFM. To analyze the change in morphology of the surface before and after the wear experiment, height scans of 100 × 100 μm^2^ in area were obtained using a Si, n-type (Si_3_N_4_) tip with an Al coating (resonant frequency f = 66 kHz, k = 3 N m^–1^, AppNano) operating in tapping mode. Root mean square roughness (RMS) values before and after wear experiments were obtained.

Macrowear experiments were performed with an established procedure of using a ball-on-flat tribometer^51^. A sapphire ball of 3 mm diameter was fixed in a stationary holder. A load of 10 mN was applied normal to the surface, and the tribometer was put into reciprocating motion. Stroke length was 6 mm with an average linear speed of 1 mm s^–1^. Surfaces were imaged before and after the tribometer wear experiment using an optical microscope with a CCD camera (Nikon Optihot-2) to examine any changes^49^.

Contact pressures for both AFM and tribometer wear experiments were calculated based on Hertz analysis^51^. The elastic modulus of PDDA^54^, 0.16 GPa, was used to estimate the elastic modulus of the composite coating, and a Poisson’s ratio of 0.5 was used (estimated). The elastic modulus of final coating is expected to be higher, so an underestimated pressure will be obtained with the selected modulus. The elastic modulus of 70 GPa and Poisson’s ratio of 0.2 were used for the borosilicate ball used in the microscale wear experiments^55^. The elastic modulus of 390 GPa and Poisson’s ratio of 0.23 were used for sapphire ball used in the macroscale wear experiments^56^. The mean contact pressures were calculated as 4.87 MPa and 2.26 MPa for the AFM (micro) and ball-on-flat tribometer (macro) experiments respectively. Microscale wear experiments were performed for 1 cycle while macroscale wear experiments were performed for 100 cycles. Therefore, the macroscale wear experiments can cause a relatively high degree of damage to the coating even though the mean contact pressures are comparable to the microscale technique.

### Self-cleaning experiment

The self-cleaning characteristics of the surfaces were examined using an experimental setup previously reported^4^. Coatings were contaminated with silicon carbide (SiC, Sigma Aldrich) in a glass chamber (0.3 m diameter and 0.6 m high) by blowing 1 g of SiC powder onto a sample for 10 s at 300 kPa and allowing it to settle for 30 min. The contaminated sample was then secured on a stage (45° tilt) and water droplets (total volume 5 mL) were dropped onto the surface from a specified height. Once dried, images were taken using an optical microscope with a CCD camera (Nikon, Optihot-2). The removal of particles by the water droplets was compared before and after tests. The ability for the water stream to remove particles was quantified using image analysis software (SPIP 5.1.11, Image Metrology A/S, Horshølm, Denmark).

### Anti-smudge experiment

The anti-smudge characteristics of the surfaces were examined using an experimental setup previously reported^30^. Coatings were contaminated as reported above. The contaminated sample was then secured on a stage and a hexadecane-impregnated microfiber wiping cloth was glued to a horizontal glass rod (radius 0.5 mm) fixed on a cantilever above the sample. As the cloth was brought in contact with the sample, the microfiber cloth was set to rub the contaminated sample under a load of 5 g for 1.5 cm at a speed of about 0.2 mm s^–1^. Images were taken using an optical microscope with a CCD camera (Nikon, Optihot-2). The removal and transfer of particles by the cloth was compared before and after tests.

### Anti-icing experiment

The anti-icing characteristics of the surfaces were examined by placing the coated samples in a freezer set at –18 °C for 2 h. The samples were tilted 10° and droplets of supercooled water (–18 °C) were then dropped onto the samples from a height of 5 cm.

### Anti-fogging experiment

The anti-fog characteristics of the surfaces were examined by placing the coated samples over boiling water for 5 s. The steam condensed on the coatings and was then photographed to determine the resulting transparency.

### Oil–water separation experiment

The superhydrophobic/superoleophilic and superhydrophilic/superoleophobic coatings were found to be suitable for oil–water separation. The stainless steel meshes (#400) were first cleaned with acetone and 2-propanol (Fisher Scientific) until they were found to be hydrophilic, then the coatings were deposited onto the meshes via spray coating. The coated meshes were then placed on top of beakers. Agitated mixtures of hexadecane and water were then poured onto the coated meshes. In separate experiments, the meshes were inclined at an angle and the oil–water mixtures were poured over them. To improve contrast, Oil Red O and Blue 1 were used as oil and water dispersible dyes respectively. The use of dyes was not found to have any effect on the performance of the coating.

### Transparency measurements

A line-of-sight light apparatus was assembled using a diffractive spectrometer (Acton, Princeton Instruments), an intensified CCD camera and an incandescent light bulb as a point source, which emitted a black-body type spectrum across the 400–700 nm bandwidth of interest. The sample slides were placed within 1–2 mm of the incandescent light source. A pair of 50-mm diameter, 100-mm focal length plano-convex lenses was used to collect emission from the light source and focus it onto the entrance slits of the spectrometer. For a single camera exposure, the spectrometer bandwidth was approximately 80 nm, so the grating was stepped at ~60 nm intervals to sample the entire bandwidth with an overlap of about 40 nm between each grating position. A single camera exposure was acquired at each grating position. The spectra were then background subtracted and divided by the spectrum acquired from an uncoated glass slide and the data plotted as a function of wavelength (400–700 nm).

## Results and Discussion

Each of the coatings comprises separate layers (total thickness ca. 630 nm) each deposited individually as shown in Fig. 2. For all coatings, the first layer comprises PDDA (thickness ca. 200 nm) and acts as an anchor layer to the glass substrate. The second layer contains SiO_2_ nanoparticles (NP, thickness ca. 350 nm) and acts as the roughness layer, enhancing the overall liquid–solid interactions. Third is a second polymer layer (PDDA (2), thickness ca. 50 nm), which helps to bind the nanoparticle layer, improving adhesion and mechanical durability. A final, functional layer (FL) is then deposited to provide the desired surface functionality. For the superhydrophilic/superoleophilic coating, there is no separate functional layer. For superhydrophobic/superoleophilic and superhydrophobic/superoleophobic coatings, the final layer is a silane layer (thickness ca. 25 nm), which condenses onto the hydrophilic PDDA (2) layer and provides either water- (methylsilane) or water- and oil-repellency (fluorosilane). For the superhydrophilic/superoleophobic coating, the final layer is a fluorosurfactant layer (thickness ca. 30 nm), which complexes with the positively charged PDDA (2) layer and provides the oil-repellency. Deposition of a separate functional layer ensures the correct functionality at the air interface without compromising the durability of the bulk coating.

### Wettability of coated surfaces

**Figure 3:**
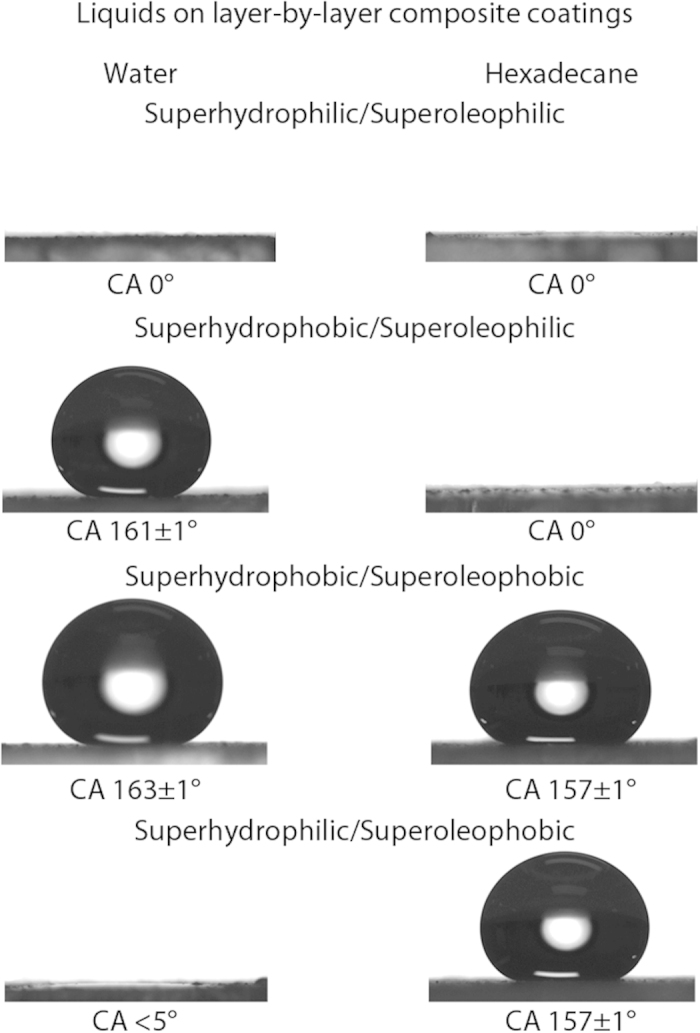
Water and hexadecane droplets (5 μL) deposited on the four layer-by-layer composite coatings.

Water and hexadecane droplet images and contact angles for all four coatings are shown in Fig. 3. The superhydrophilic/superoleophilic coating was instantly wet by both water and oil. The superhydrophobic/superoleophilic coating was wet by oil whilst repelling water. The superhydrophobic/superoleophobic coating repelled both liquids. Finally, the superhydrophilic/superoleophobic coating repelled oil but was wet by water. Table 3 provides a summary of all contact angle data.

For both superoleophobic coatings, hexadecane contact angles were found to be above 150° with tilt angles <5°, whilst for both superhydrophobic coatings, water contact angles were above 160° with tilt angles <2°. This suggests the formation of a composite air/solid interface and that droplets were in the Cassie-Baxter regime. Oil repellency of both superoleophobic coatings has been further tested in previous work^46^,^47^. The coatings were found to remain superoleophobic for tetradecane, dodecane, decane, and octane; with only slight increases in tilt angles for the lower chain length oils, due to their lower surface tensions.

The oil repellency of the superhydrophilic/superoleophobic coating, in addition to wetting by water, is due to the fluorosurfactant containing a low surface tension fluorinated tail and a high surface tension head group complexed with a hydrophilic polyelectrolyte, shown in Fig. 2. During spray coating, the polar head group forms an electrostatic complex with the polyelectrolyte layer below and the fluorinated tails orient themselves at the air interface. Large, bulky oil molecules are trapped at this fluorinated interface while smaller water molecules can more easily penetrate down through the thin layer (ca. 30 nm) to the hydrophilic region where the surfactant head group complexes with the polyelectrolyte layer^40^,^42^. The result is a “flip-flop” of surface properties and a coating that repels oils but is wet by water, Fig. 1. Water droplets (5 μL) were found to immediately (less than 2 s) wet the surface in contrast to previous work where water penetration can take 5–30 min^34^,^38^,^39^,^41^ and similar behavior was found for both larger and smaller droplets. This is due to the fluorosurfactant only being present as a single layer at the air interface allowing water to wick down to hydrophilic polyelectrolyte layer beneath. This instant affinity for water is a big advantage over other techniques in various applications such as anti-fogging and oil–water separation where it is crucial the water spreads out as quickly as possible.

### Wear resistance of coated surface

**Figure 4:**
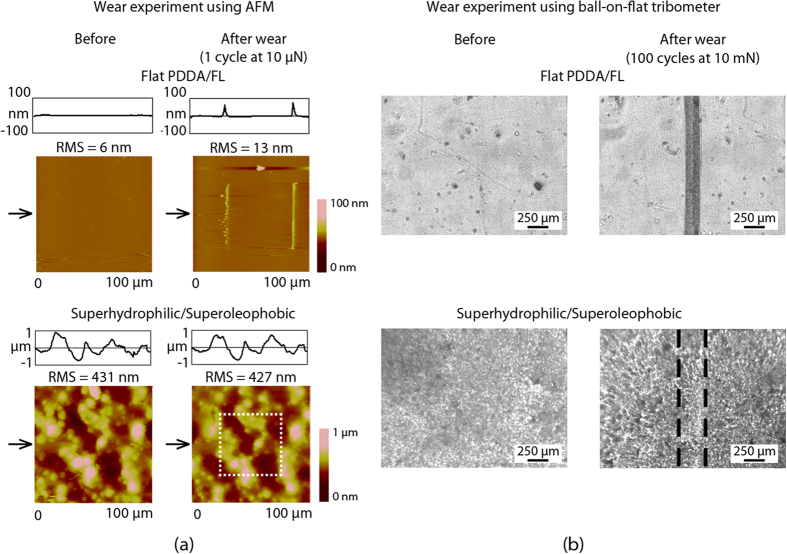
(**a**) Surface height maps and sample surface profiles (locations indicated by arrows) before and after AFM wear experiment with 15 μm radius borosilicate ball at a load of 10 μN for flat and superhydrophilic/superoleophobic layer-by-layer composite coatings. RMS roughness values are displayed, and (**b**) optical micrographs before and after wear experiments using ball-on-flat tribometer at 10 mN for flat and hydrophilic/oleophobic layer-by-layer composite coatings. Similar results were obtained for the three remaining layer-by-layer composite coatings.

The mechanical durability of the coatings was investigated through the use of AFM and tribometer wear experiments and the resulting images are shown in Fig. 4. AFM images show a 100 × 100 μm^2^ scan area with the wear location (50 × 50 μm^2^) in the center of each image. The optical images show a portion of the wear track from the tribometer experiments. For the soft PDDA/FL coating (ca. 225 nm thick), there is significant wear with both AFM and tribometer experiments causing observable damage to the surface. In contrast, the layer-by-layer composite coating survived the AFM wear experiment with no observable defects. For the tribometer experiment, there is some noticeable burnishing to the coating, however it is minimal when compared to the PDDA/FL coating. Higher magnification images confirmed that the layer-by-layer composite coating morphology is similar before and after the wear test and there is no removal of the coating from the substrate. This is in contrast to the PDDA/FL coating, which was completely destroyed by the wear test to reveal the substrate underneath. Similar results were found for the other three coatings investigated here. This suggests that the hard SiO_2_ nanoparticle layer (underneath ca. 75 nm thick PDDA/FL layers) helps improve the durability of the coating, while the oppositely charged PDDA binder layers help anchor the particles to the glass substrate via an electrostatic bond. This is in contrast to other polymer-nanoparticle coatings where the interfacial adhesion is not aided by this electrostatic attraction.

For both superoleophobic layer-by-layer composite coatings, previous work has demonstrated that they are both able to maintain their oil-repellency after wear testing, with hexadecane droplets rolling over and from the wear scar with little to no impediment^46^,^47^. Additionally, superhydrophilic/superoleophobic coated samples kept in storage for ca. 9 months were found to retain their surface properties.

To further demonstrate the benefits of the layered structure on the mechanical durability of the coating, a fluorosurfactant-containing, superhydrophilic/superoleophobic coating was fabricated using a “one-pot” technique, where all the materials were mixed (at the same concentrations used in the layer-by-layer technique) and deposited together. This coating, which was found to be similar in terms of thickness and roughness as the layer-by-layer composite coating, was then subjected to the same ball-on-flat tribometer experiment as described above. The coating was found to have significantly poorer adhesion to the glass substrate than the layer-by-layer composite coating, most likely due to the presence of the low surface tension material throughout the coating instead of solely at the air interface as in the layer-by-layer composite coating.

Finally, depending upon the functional layer used, the coatings will display thermal stabilities from 65–175 °C with both the methylsilane (66 °C) and fluorosilane (85 °C) displaying lower thermal stabilities than the fluorosurfactant layer (175 °C).

### Transparency of coated samples

**Figure 5:**
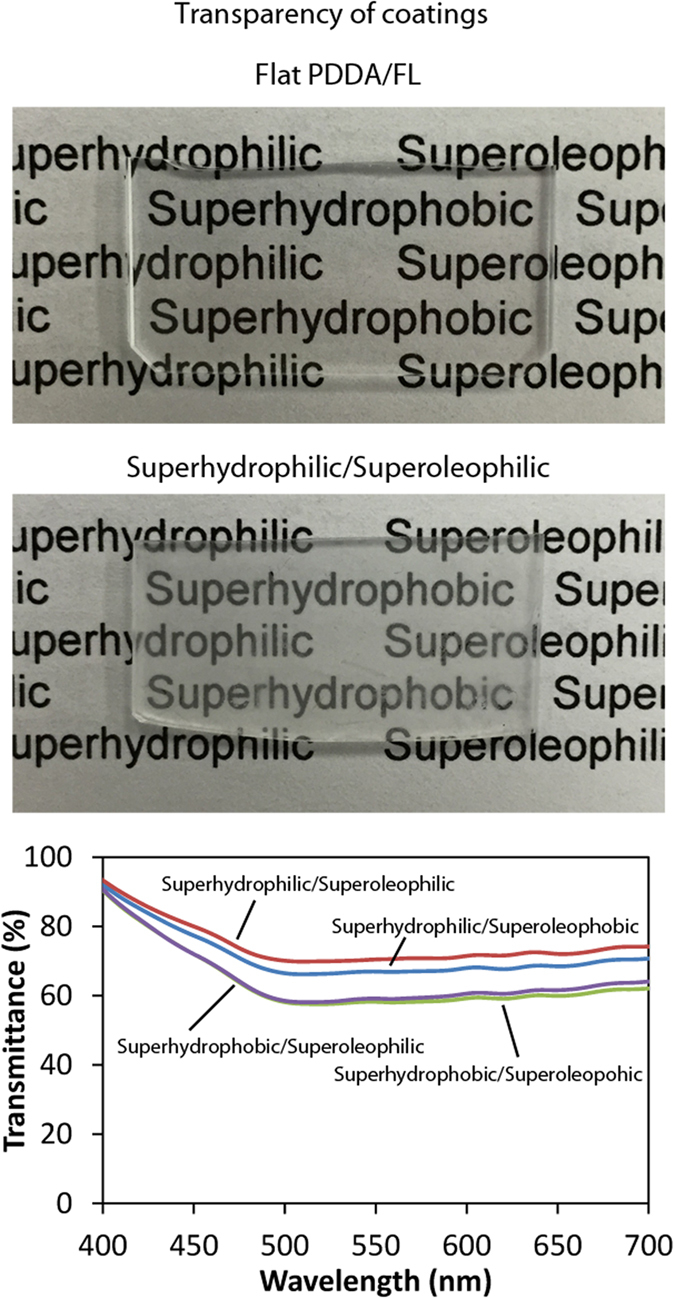
Photographs of flat and superhydrophilic/superoleophobic layer-by-layer composite coatings. The flat coating appears transparent. Any reduction in transparency for the composite coating compared to the flat coating is due to the NP and FL layers.

Many applications of self-cleaning, anti-smudge surfaces rely on the transparency of the coating. When placed directly behind the layer-by-layer composite coating sample, text remains legible, suggesting that the coating displays characteristics of transparency, as shown in Fig. 5. The transmission of visible light through the coatings was found to vary between 58–93% of that of uncoated glass depending upon the wavelength and the specific coating. The superhydrophilic/superoleophilic coating was the most transparent with transmittance of 70–93% over the visible spectrum. A level of 70% visible light transmittance is acceptable for certain automotive applications^57^. Further improvement in transparency, potentially by decreasing the thickness of the NP and FL layers or reducing particle agglomeration, will be investigated in the future.

### Anti-fogging property of coated samples

**Figure 6:**
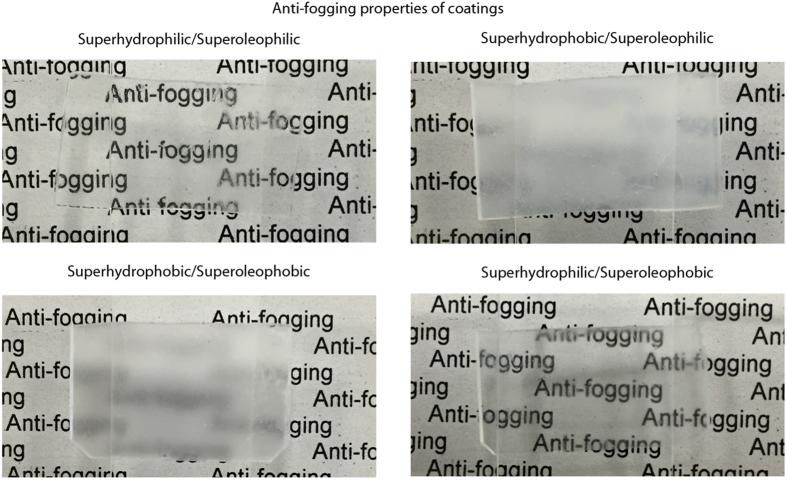
Photographs of the four layer-by-layer composite coatings after exposure to water vapor. The hydrophilic coatings maintain transparency due to the formation of a thin water film on the surface. The hydrophobic coatings become opaque due to the formation of discrete water droplets on the surface.

To examine the anti-fogging properties, all four coatings were placed directly above a source of boiling water for 5 s. The samples were then photographed to assess their transparency, shown in Fig. 6. Both the superhydrophilic coatings were found to retain their transparency with text remaining visible through the condensed water layer. In contrast, on the superhydrophobic coatings, the formation of discrete droplets of water results in samples that are completely opaque.

For the superhydrophilic/superoleophobic coating, the speed of the water penetration through the low surface tension fluorinated tail groups to the high surface tension head groups is crucial for the condensed droplets to spread out and form a continuous water layer and thereby maintain transparency. Previously developed coatings would not be suitable for anti-fogging applications because the rate of water penetration is too low (takes 5–30 min for surface to become superhydrophilic)^34^,^38^,^39^,^41^.

### Anti-icing property of coated samples

**Figure 7:**
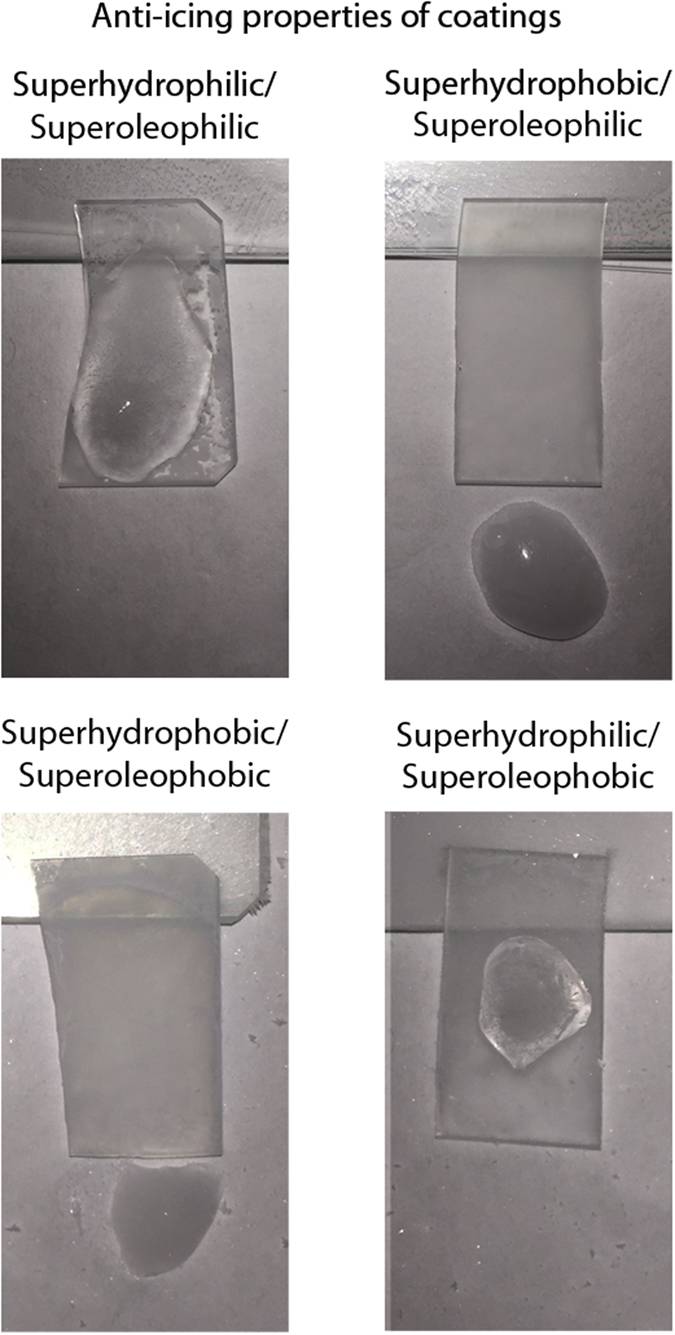
Photographs of the four layer-by-layer composite coatings after freezing and deposition of supercooled water. The water immediately froze upon contact with the hydrophilic coatings whilst the droplets were able to roll off the hydrophobic coatings before freezing.

For anti-icing experiments, all four coatings were placed in a freezer set at −18 °C for 2 h. The samples were tilted and droplets of supercooled water were deposited onto them, as shown in Fig. 7. For the superhydrophilic coatings, the droplets spread out and froze on the sample surface. For the superhydrophobic coatings, droplets rolled off the surface to freeze on the bottom of the freezer. This occurs because the water droplets are in the Cassie-Baxter state. The formation of a composite interface minimizes the contact with the cooled substrate and ensures a low hysteresis so droplets can roll from the tilted surface. This quick experiment demonstrates the potential for these coatings in anti-icing applications. Further work needs to be carried out to assess the true effect of these coatings on ice adhesion and repellency.

### Self-cleaning property of coated samples

**Figure 8:**
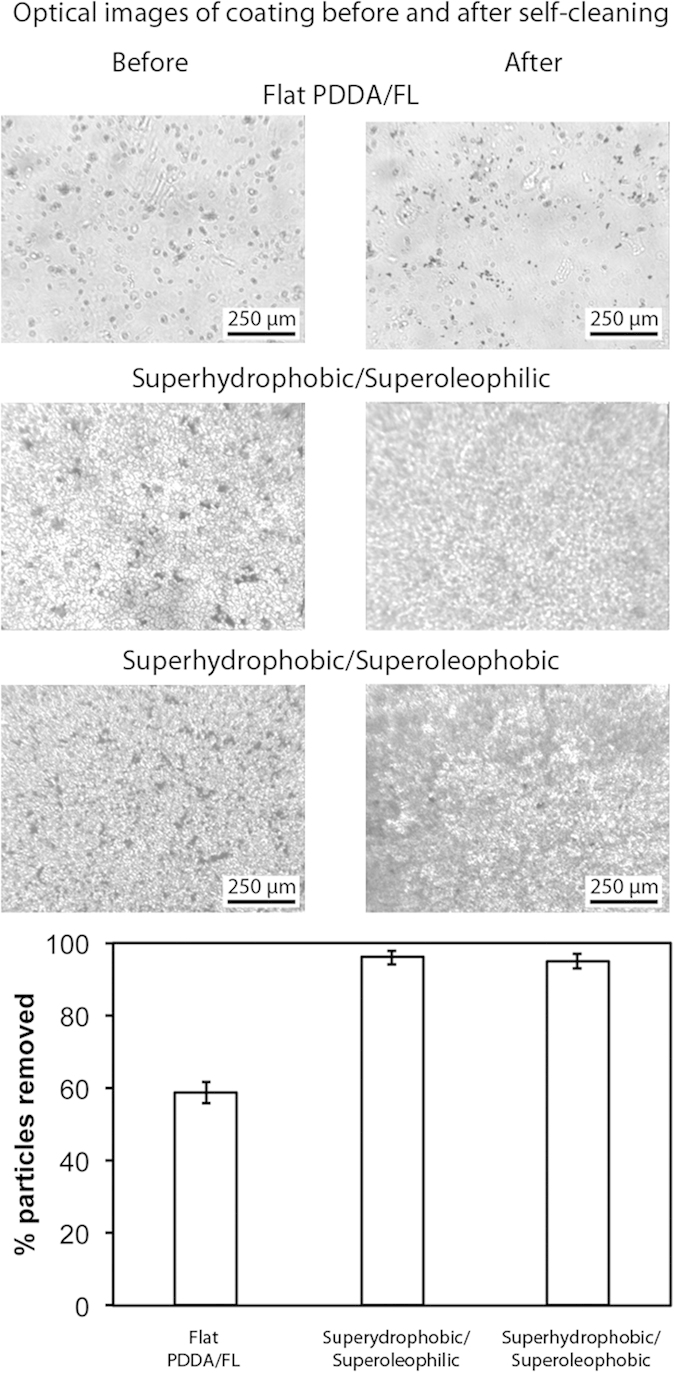
Optical micrographs of contaminated coatings before and after self-cleaning test on flat and the superhydrophobic layer-by-layer composite coatings. Dark spots on coatings and cloth indicate silicon carbide particle contaminants. Image analysis suggests a >90% removal of particles on the two composite coatings.

To examine the self-cleaning properties, the coatings were contaminated with silicon carbide particles, shown in Fig. 8. A stream of water droplets was then used to clean the surface. On the flat PDDA/FL coating this resulted in an incomplete removal of the particles with the surface remaining contaminated. For the superhydrophobic/superoleophilic and superhydrophobic/superoleophobic coatings, the vast majority of the particles were removed by the action of water droplets rolling across the repellent surfaces, collecting particles in the process. These superhydrophobic coatings are self-cleaning due to their high water contact angle and low hysteresis. Water droplets deposited onto these samples are able to roll over the coating with little impediment, collecting less hydrophobic contaminants as they go.

### Anti-smudge property of coated samples

**Figure 9:**
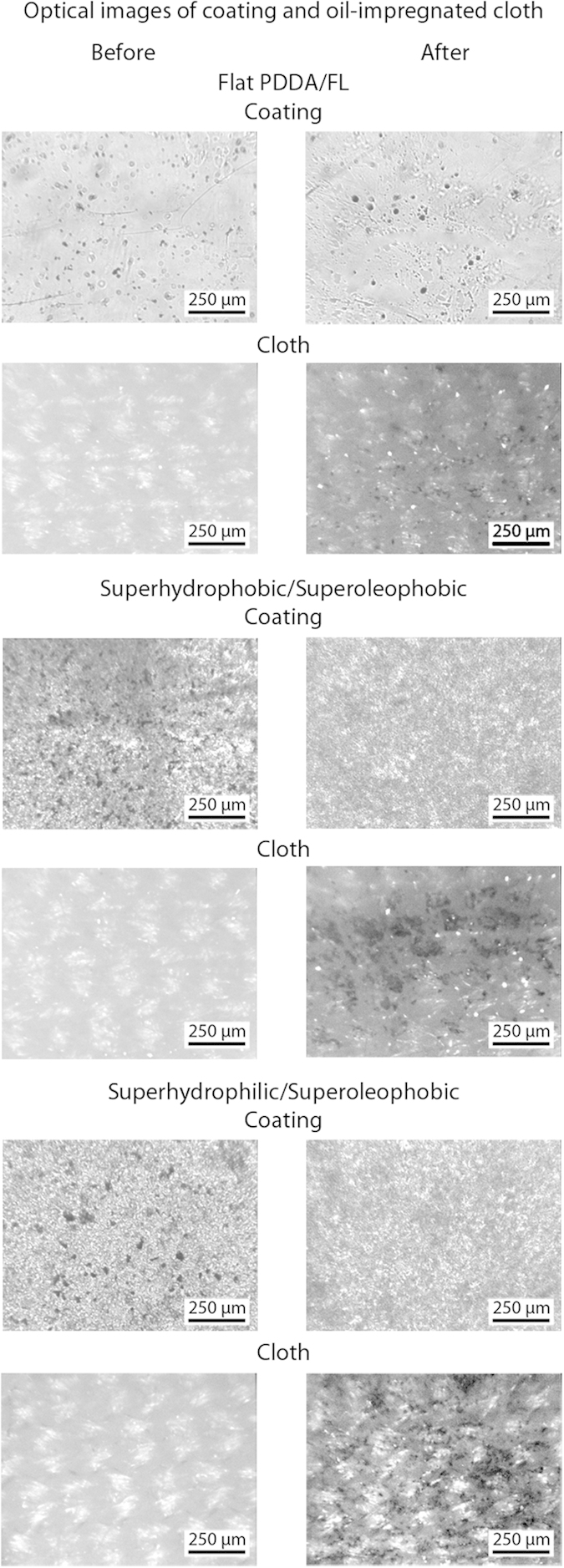
Optical micrographs of contaminated coatings and oil-impregnated microfiber cloth before and after smudge test on flat and the superoleophobic layer-by-layer composite coatings. Dark spots on coatings and cloth indicate silicon carbide particle contaminants.

To examine the anti-smudge properties of the superhydrophobic/superoleophobic and superhydrophilic/superoleophobic coatings, a hexadecane-soaked cloth was used to wipe the contaminated surfaces, shown in Fig. 9. On the flat PDDA/FL coating this resulted in incomplete removal of the particles with the surface remaining contaminated. For the oil-repellent coatings, the particles were transferred to the cloth with no observable particles remaining on the surfaces. Similarly to the self-cleaning experiments with water, the anti-smudge property relies on a high contact angle and low hysteresis for the oil. The oil in the cloth is able to collect oleophilic contaminants from the surface of the coating without sticking to the surface.

### Oil–water separation ability of coated samples

**Figure 10:**
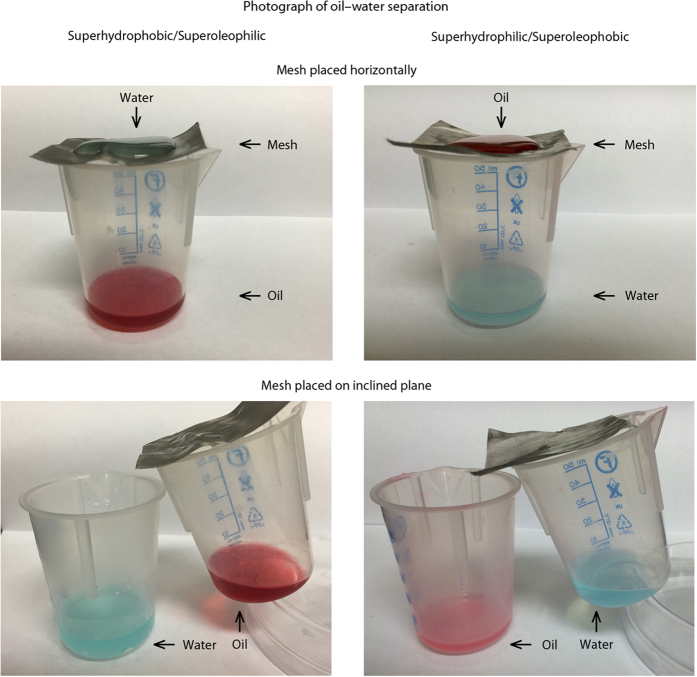
Photographs of the hydrophobic/oleophilic and hydrophilic/oleophobic layer-by-layer composite coated stainless steel meshes acting as oil–water separators. On the superhydrophobic/superoleophilic coated mesh, water collects on top of the mesh whilst oil passes through. In contrast, on the superhydrophilic/superoleophobic coated mesh, water passes through the mesh while the oil remains on the top surface. Alternatively the meshes can be placed at an angle and oil and water collected simultaneously in separate beakers. Oil and water dyes used to enhance contrast.

The superhydrophobic/superoleophilic and superhydrophilic/superoleophobic coatings exhibit different responses to water and oil and therefore are suitable for use as oil–water separators. Agitated oil–water mixtures were poured onto coated meshes suspended over beakers, as shown in Fig. 10. For the superhydrophobic/superoleophilic-coated mesh, the oil component of the mixture passed through whilst the water remained on top. Meanwhile, for the superhydrophilic/superoleophobic-coated mesh, the opposite occurred with the water component passing through the mesh and the oil remaining on top. In both cases, the liquid remaining on top of the coated mesh could be easily removed by tilting. Placing both the meshes on an inclined plane resulted in the simultaneous collection of oil and water in two separate beakers. For the superhydrophilic/superoleophobic-coated mesh, this tilted setup is only possible due to the fast penetration by water. Previously developed coatings would not be suitable for this method of oil–water separation because the rate of water penetration is too low (takes 5–30 min for surface to become superhydrophilic)^34^,^38^,^39^,^41^.

In both cases, the agitated mixture was effectively separated into the two component liquids. Discrete droplets (of water or oil, depending upon the coating used) of various sizes could be repelled, though the smallest droplet that it is possible to separate is dependent upon the mesh aperture. These coatings could be applied to different materials like meshes or filters, depending upon the application, which will determine the size of oil droplets or other organic material (for instance algae or other microorganisms) that can be removed from the water. For bulk cleanup like at an oil spill, coarse separators could be used to remove the vast majority of the oil, followed downstream by finer filters to remove smaller contaminants.

These proof of concept experiments demonstrate that superhydrophobic/superoleophilic and superhydrophilic/superoleophobic coatings could find use in oil–water separation applications, however further work is required to determine their full effectiveness and suitability in real world applications.

## Conclusions

A fabrication technique has been developed that can be used to create coatings with four possible combinations of water and oil repellency and affinity. These coatings have been fabricated through the use of a novel combination of deposition techniques utilizing the charged layer-by-layer method for durability plus the addition of a functional layer on top for the desired surface properties. The superoleophobic coatings display oil contact angles of >150° and tilt angles <5° and the superhydrophobic coatings display water contact angles of >160° and tilt angles <2°. One coating combines both superoleophobic and superhydrophobic properties whilst others can be used to mix and match oil and water repellency and affinity.

The coatings are found to be mechanically durable with micro- and macrowear experiments not causing any considerable damage due to the hard SiO_2_ nanoparticles and the electrostatic interaction between the base layers. Additionally, these surfaces were found to display characteristics of transparency with an averaged transmission of 75% and text remaining visible through the coating. This level of transparency is acceptable for certain automotive applications.

The applications of the coatings are dependent upon the functional layer used. Superhydrophilic/superoleophilic coatings could find use in anti-fogging. Superhydrophobic/superoleophilic coatings could be used for self-cleaning, anti-fouling, anti-icing, and oil–water separation. The superhydrophobic/superoleophobic coating is suitable for self-cleaning, anti-fouling, anti-smudge, and anti-icing.

Finally, the superhydrophilic/superoleophobic coating could be used for anti-fouling, anti-smudge, anti-fogging, and oil–water separation. This particular coating could be useful in anti-biofouling, where superoleophobicity, superhydrophilicity and nanostructuring all contribute to reducing microorganism attachment. Additionally, when applied to a porous substrate, this coating was found to separate oil from water. These coatings, which are produced from non-toxic materials, could also help reduce the environmental impact of the gas, oil, metal, textile, and food-processing industries.

## Additional Information

**How to cite this article**: Brown, P. S. and Bhushan, B. Bioinspired, roughness-induced, water and oil super-philic and super-phobic coatings prepared by adaptable layer-by-layer technique. *Sci. Rep*. **5**, 14030; doi: 10.1038/srep14030 (2015).

## Figures and Tables

**Table 1:** Examples of past coatings from the literature for each combination of water and oil repellency and affinity. No method covers all four.

Surface properties	Materials	Contact/tilt angles	Comments	Ref
Hydrophilic/Oleophilic	Dip coated poly(allylamine hydrochloride) (PAH), poly(sodium 4-styrene sulfonate) (PSS), silica nanoparticles	Water: ca. 0° Oil: N/A	Layer-by-layer technique used for anti-fogging coatings. No durability testing	8
	Dip coated PDDA, PSS, silica nanoparticles	Water: ca. 0° Oil: N/A	Superhydrophilic, anti-reflective coatings. No durability testing	9
	Dip coated polydimethyldiallyl- ammonium chloride (PDDA), PSS, silica nanoparticles	Water: ca. 0° Oil: N/A	Layer-by-layer used to create hydrophilic channels for microfluidics. No durability testing	11
Hydrophobic/Oleophilic	Spray coated PTFE	Water: 156° Diesel oil: <4°	Applied to a mesh for oil–water separation. No durability testing	13
	Spray coated acrylic polymer and organosilane modified silica particles	Water: ca. 160°/ ca. 1 Oil: N/A	Applied to aluminum plates for anti-icing. No durability testing	17
	Spray/spin coated fluoropolymer, nanoparticles	Water: 153°/8° Oil: N/A	Applied to aluminum plates for anti-icing. No durability testing	18
	Dip coated polystyrene, polydimethylsiloxane modified silica nanoparticles	Water: 157°/4° Oil: ca. 0°	Applied to filter paper for oil–water separation. No durability testing	14
	Chemical vapor deposition (CVD) of carbon nanotubes	Water: 150°/7° Gasoline: 0°	Applied to mesh for oil–water separation. No durability testing	15
	Spray coated/sandblasted PTFE	Water: 153° Oil: N/A	Applied to aluminum plates for anti-icing and ice adhesion tests	19
Hydrophobic/Oleophobic	Spray coated perfluoroalkyl methacrylic copolymer, TiO_2_ nanoparticles	Water: 164° Ethylene glycol: 144°	Hysteresis not studied, only ethylene glycol investigated. No durability testing	21
	CVD fluorosilane on textured, re-entrant SiO_2_	Water: N/A Octane: 163°/18°	No durability testing. Complex texturing required	22
	Photolithography then electropolymerization of tetrabuty- lammonium hexafluorophosphate	Water: 160° Hexadecane: 144°/40°Dodecane: 135°	Poor oil repellency (high tilt angles). No durability testing	24
	Spin coated PDMS, silica nanoparticles then dip coated perfluorooctyl trichlorosilane	Water: 153°/ca. 0° Diiodomethane: 141°/12°	Coating shows reasonable durability. No low surface tension liquid repellency testing	25
	Spray coated perfluorooctanoic acid, copper acetate	Water: 163°/4° Rapeseed oil: 155°/10°	Poor oil repellency (high tilt angles) and durability	28
	Silica aerogel plus fluorinated surfactant	Water: 172°/22° Paraffin oil: 168°/38°	Poor repellency (high tilt angles) but good durability	29
	Electrospray poly(dimethylsiloxane), fluorodecyl polyhedral oligomeric silsequioxane	Water: ca. 160°/2° Hexadecane: ca. 150°/5°	No durability testing	26
	Dip coated silicone resin, silica nanoparticles then CVD of fluorosilane	Water: 170°/1° Hexadecane: 153°/4°	Good repellency and durability. Only tested on PET	31
Hydrophilic/Oleophobic	Synthesis of fluoroalkylated silicon co-oligomers	Water: 72° Water (after 25 min): 0° Dodecane: 60°	Coating is initially hydrophilic, not superhydrophilic as intended. Poor oil repellency. No durability testing	34
	Plasma deposited polymer then dip coated fluorosurfactant	Water: <20° Hexadecane: 82°	Not water affinity and oil repellency. No durability testing	36
	Synthesis of dimethylacrylamid fluoroalkyl end-capped oligomers, silica gel hybrids	Water: 48° Water (after 30 min): 0° Dodecane: 41°	Coating is initially hydrophilic, not superhydrophilic as intended. Poor oil repellency. No durability testing	38
	Spray coated PDDA, sodium per- fluorooctanoate, silica nanoparticles	Water: 165° Water (after 9 min): 0° Hexadecane: 155° Dodecane: 152°/10°	Coating is initially superhydrophobic, not superhydrophilic as intended. Requires additional treatment not suitable for industry. No durability testing	39
	Spin/dip coated poly(styrene–*co–*maleic anhydride), fluorosurfactant	Water: <10° Hexadecane: 112°	Poor oil repellency. No durability testing	40
	Dip coated fluoroalkyl end-capped vinyltrimethoxysilane oligomer, CaSi_2_ particles	Water: 129° Water (after 5 min): 0° Dodecane: 118°	Coating is initially hydrophobic, not superhydrophilic as intended. Poor oil repellency. No durability testing	41

**Table 2:** Comparison of the various applications of the four layer-by-layer composite coatings.

Functional layer	Surface properties	Applications
None	Superhydrophilic/Superoleophilic	Anti-fogging
Silane	Superhydrophobic/Superoleophilic	Self-cleaning Anti-fouling Anti-icing Oil–water separation
Fluorosilane	Superhydrophobic/Superoleophobic	Self-cleaning Anti-fouling Anti-smudge Anti-icing
Fluorosurfactant	Superhydrophilic/Superoleophobic	Anti-fouling Anti-smudge Anti-fogging Oil–water separation

**Table 3:** Comparison of contact and tilt angles for water and hexadecane droplets deposited on the four layer-by-layer composite coatings.

Coating	Water		Hexadecane	
	Contact angle (°)	Tilt angle (°)	Contact angle (°)	Tilt angle (°)
Superhydrophilic/Superoleophilic	∼0	N/A	∼0	N/A
Superhydrophobic/Superoleophilic	161 ± 1	2 ± 1	∼0	N/A
Superhydrophobic/Superoleophobic	163 ± 1	2 ± 1	157 ± 1	4±1
Superhydrophilic/Superoleophobic	<5	N/A	157 ± 1	4±1
